# The Accumulation and Biosynthesis of Anthocyanin in Black, White, and Yellow Waxy Corns (*Zea mays* L. *sinensis kulesh*) during Kernel Maturation

**DOI:** 10.3390/foods12071486

**Published:** 2023-04-01

**Authors:** Xiaodan Hu, Jianhua Liu, Qiji Shan, Song Bai, Wu Li, Tianxiang Wen, Xinbo Guo, Jianguang Hu

**Affiliations:** 1 Key Laboratory of Crops Genetics Improvement of Guangdong Province, Crop Research Institute, Guangdong Academy of Agricultural Sciences, Guangzhou 510640, China; 2 School of Food Science and Engineering, South China University of Technology, Guangzhou 510640, China; 3 Guangdong Rice Engineering Laboratory, Rice Research Institute, Guangdong Academy of Agricultural Sciences, Guangzhou 510640, China

**Keywords:** *Zea mays* L. *sinensis kulesh*, waxy corn, phenolic, anthocyanin, antioxidant activity, biosynthesis

## Abstract

Waxy corn kernels with different colors have high phenolic content and good application potential in medicine and food healthcare. In our work, the content changes of phenolic and anthocyanins profiles were related to genes in the anthocyanin biosynthesis pathway, and the antioxidant activities of three different colors of waxy corn kernels (black, white, and yellow) were determined during kernel development. Results showed that growing temperature and light intensity could affect the accumulation of phytochemicals and antioxidant activities in waxy corns during maturation. Phenolic and antioxidant activities decreased over kernel maturation, and spring had higher nutrition levels during the best harvest time (20 and 25 days after pollination in the spring and autumn, respectively) for waxy corns. Cyanidin-3-*O*-glucoside and pelargonidin-3-*O*-glucoside were the main anthocyanins detected in the black waxy corns. The contents of cyanidin are higher than pelargonidin followed by peonidin in the autumn, while on the other hand, pelargonidin had a slightly higher content compared to cyanidin in the spring. *DFR*, *CF1*, and *ANS* were the key genes affecting anthocyanin accumulation. This work provided information on the best harvest time for the pigment of waxy corn in order to achieve relatively high phenolic profiles and antioxidant activities. It also illustrated the possible relationship between weather conditions, gene expression levels, and phenolic content during kernel development.

## 1. Introduction

Waxy maize was first found in the south area of China and it has a long cultivation history all over the world. Compared to normal maize, it has a much higher viscosity, sticky taste, and higher digestibility because of its high amylopectin content [1]. Apart from the difference in taste and amylopectin, waxy corn also has a higher content of protein, folic acid, carotenoids, and phenolics compared to normal maize [2]. It has abundant grain colors varying from white, red, purple, yellow, black, and so on, which is mainly caused by different contents of phytochemicals, including phenolics, carotenoids, etc. In waxy corn kernels, a phenolic is one of the important antioxidants because of its chemical structures, including the degree of glycosylation and the hydroxyl group that could be easily oxidized and have strong scavenging ability to free radicals, as well as metal chelating activity [3]. It can protect cells from oxidative damage and, therefore, has anti-inflammatory, anti-cancer, and anti-diabetic functions [4]. Pigmented waxy maize has abundant anthocyanin content compared to normal waxy maize and it is usually accumulated in the aleurone and pericarp layer of the kernels [5]. Cyanidin, peonidin, and pelargonidin were the most common anthocyanins reported in pigmented waxy corn kernels [6]. Anthocyanin has a positively charged oxygen atom and it is one of the strongest antioxidants in nature and it has a higher antioxidant capacity of about 20 times and 50 times of vitamin C and vitamin E, respectively [7,8]. In addition, anthocyanin is also a safe and non-toxic food pigment that has good application potential and research value in medicine and food healthcare.

Anthocyanin biosynthesis belongs to the branch of phenylpropanoid and flavonoid pathways and involves the effect of many structural genes, transcription factors, and environmental factors [7]. The first phase of the biosynthesis pathway is shared with many other secondary metabolites. The phenylalanine formed from the shikimic acid pathway is catalyzed by phenylananine lyase (PAL) to generate cinnamic acid, and then under the catalysis of cinnamate-4-hydroxylase (C4H) and 4-coumarate: CoA ligase (4CL) to form 4-Coumaroyl-CoA. The second phase of biosynthesis is the key synthesis reaction of flavonoids. Naringenin was catalyzed by chalcone synthase (CHS) and chalcone isomerase (CHI). Afterward, it could be turned into dihydrokaempferol by flavanone 3-hydroxylase (F3H). Dihydroquercetin and dihydromyricetin were formed in the presence of flavanone 3′-5′-hydroxylase (F3′5′H) and flavonoid 3′-hydroxylase (F3′H), respectively. All three substances could then be catalyzed by dihydroflavonol 4-reductase (DFR) and anthocyanin synthetase (ANS) to form anthocyanin. These unstable anthocyanins need to be stabilized via UDP-glucose: flavonoid 3-*O*-glucosyltransferase (UFGT) before transportation to the cell vacuole for storage [9].

The varieties of phenolic and antioxidant activity in matured waxy corns have been widely studied [10,11,12]. However, the phenolic and anthocyanin content changes during the maturation process of pigment waxy corns under two different weather conditions, which have not been investigated in detail. Additionally, the changes in expression levels of key genes were rarely studied to interpret the changes in anthocyanin content during kernel maturation. Our research focuses on the phenolic accumulation and antioxidant activity changes of waxy corns during kernel maturation over two growing seasons by analyzing the phenolic composition, in vitro and cell antioxidant activity, and the expressions of relative genes involved in the biosynthesis of phenolics and anthocyanins. Weather conditions were also taken into consideration to interpret the changes of anthocyanin during kernel maturation because anthocyanin biosynthesis may be affected greatly by light, temperature, and water stress [13]. This work provides an overview of phenolic and anthocyanin changes in various colors of waxy maize kernel during maturation in different weather conditions and also lays the foundation for future harvest advice of colored waxy corns. We studied the function of key candidate genes responsible for anthocyanin accumulation in black waxy corns.

## 2. Materials and Methods

### 2.1. Plant Materials and Reagents

Black (L1), white (L2), and yellow (L3) waxy corns were cultivated in the same field with standardized planting and management in two growing seasons. Kernels that were similar in shape and disease-free were collected from 10 to 25 days after pollination (DAP) in the spring and from 15 to 30 DAP in the autumn with a five-day sampling frequency. A biological triplicate of kernels was collected and frozen by liquid nitrogen. Weather conditions during cultivation were recorded, including the average temperature (°C), relative humidity (RH, %), rainfall (mm), and average photosynthetically active radiation (PAR, μmol/m^2^·s).

Phenolic (gallic acid, ferulic acid, lutin, *p*-coumaric acid, and epicatechin) and anthocyanin standards (cyanidin-3-*O*-glucoside, pelargonidin-3-*O*-glucoside, cyanidin, pelargonidin, and peonidin) were purchased from Weikeqi Co., Ltd. (Chengdu, Sichuan, China). High-performance liquid chromatography (HPLC) reagents were purchased from ANPEI (Shanghai, China). A human liver cancer cell (HepG2, ATCC^®^ HB-8065) was purchased from ATCC company (Manassas, VA, USA) and the cell culture reagents including Williams medium E (WME), fetal bovine serum (FBS), Trypsin-EDTA, and insulin were purchased from GIBCO Life Technologies (Grand Island, NY, USA).

### 2.2. Extraction, Qualitative and Quantitative Analysis of Phenolic and Anthocyanin

Phenolic extracts were obtained referring to the method reported previously with modification [14]. Briefly, 5 g of grounded samples were extracted with 35 mL of chilled acidified 80% acetone (pH = 2, acidified with HCl). Then, the supernatants were collected by centrifuging under 8000 revolutions per minute (rpm) for 10 min. This extraction process was repeated 3 times. The supernatants were evaporated to dryness at 45 °C using a rotary evaporator under a vacuum. Residues were collected by redissolving with 10 mL of 75% methanol and were stored at −20 °C until used. The residues after extractions with acetone were digested by 20 mL of 2 M sodium hydroxide for 2 h while shaking and then they were adjusted to pH = 2 with HCl. The bound phenolic extracts were extracted with ethyl acetate 3 times. The supernatants were collected by centrifugation and evaporated until dryness. The residues were also resolved by 75% methanol. Biological triplicates were performed.

Phenolics and anthocyanins were analyzed using HPLC reported previously with modification [15]. HPLC conditions were listed as follows. A Waters C18 column (250 × 4.6 mm, 3.5 μm) was used under a testing temperature of 35 °C and a flow rate of 1.0 mL/min. A Waters photodiode array detector (Waters 2998, Waters Corporation, Milford, MA, USA) was used as the detector under a wavelength of 280 nm and 520 nm. The mobile phases were A: 0.1% trifluoroacetic acid (A) and B: acetonitrile (B). Gradient elution was 0–5 min (90% phase A), 5–17 min (90–78% phase A), 17–22 min (78–75% phase A), 22–30 min (75–65% phase A), 30–34 min (65–42% phase A), 34–40 min (42–10% phase A), 40–50 min (10–90% phase A), and 50–60 min (90% phase A). Results were shown as milligrams per 100 g of dry weight (DW) (mg/100 g DW) (mean ± SD, *n* = 3).

However, there were plenty of varieties of anthocyanin with glucosides, and it was difficult to analyze all the anthocyanins by comparing them with anthocyanin standards using HPLC. The quantification of anthocyanin is not accurate under this circumstance. Hence, the hydrolysis reaction was conducted to achieve anthocyanin without glucoside attached in order to have detailed information for the quantification of each type of anthocyanin. Briefly, 1 mL of extracts were evaporated to dryness under nitrogen and dissolved with 1 mL of acidified water (pH = 2). Then, the extracts were sealed and heated in boiling water for 1 h to remove glucoside. Biological triplicates were performed. HPLC was used to analyze anthocyanin. The HPLC conditions were the same as described above and the wavelength was 520 nm. The data were expressed as milligrams per kilogram DW (mg/kg DW) (mean ± SD, *n* = 3).

### 2.3. In Vitro and Cell Antioxidant Activity Analysis

In vitro antioxidant activity of free and bound phenolic extracts was tested using an oxygen radical absorbance capacity (ORAC) assay [16]. Briefly, samples were diluted using a phosphate buffer working solution before adding to a 96-well plate. A fluorescein working solution was added and incubated for 20 min at 37 °C. The data were tested after adding a 2,2′-azobis(2-amidinopropane)dihydrochloride (ABAP) solution. Trolox solutions were also tested as standards. The final results were expressed as μmol Trolox equivalent per gram DW (μmol TE/g DW) (mean ± SD, *n* = 3).

Cellular antioxidant activity (CAA) was used to test cell antioxidant activities [17]. Cell antioxidant activity includes phosphate buffered saline (PBS) wash and no PBS wash protocols. Briefly, HepG2 cells were incubated at 37 °C in 5% CO_2_ to attach to the plate. Various concentrations of water-soluble extracts and fluorescent substances were added to the cells in the growth medium and incubated for 1 h. After incubation of cells with extracts, the treatment mediums were removed and a working solution with free radicals were added (no PBS wash protocol), or the mediums were removed and wash again with 100 μL PBS before adding a working solution with free radicals (PBS wash protocol). Results were tested using FilterMax F5 Multi-Mode Microplate Reader (Molecular Devices, LLC, San Jose, CA, USA). The data were expressed as μmol quercetin equivalent per 100 g DW (μmol QE/100 g DW) (mean ± SD, *n* = 3).

### 2.4. Analysis of Gene Expression during the Anthocyanin Biosynthesis Pathway by a Real-Time Quantitative Polymerase Chain Reaction (RT-qPCR)

An HP Plant RNA Kit (OMEGA, Norcross, GA, USA) was used to extract RNA. Then, the RNA was reversely transcribed to cDNA by a fastking gDNA dispelling RT Supermix kit (TIANGEN, Beijing, China). RT-qPCR tests were conducted by a Roche LightCycler^®^ 480 (F. Hoffmann-La Roche Ltd., Basel, Switzerland) with a SuperReal PreMix Plus (SYBR Green) kit (TIANGEN, Beijing, China). *ADF* was the reference gene, and all the gene IDs and primers were listed in Supplementary File. Relative gene expression levels were expressed as mean ± SE, with cycle threshold (Ct) values calculated by the 2^−ΔΔCt^ method.

### 2.5. Statistical Analysis

Data and graphs were analyzed and plotted by Origin 2018 (Origin Lab Corporation, Northampton, MA, USA). Statistical differences were analyzed by one-way analysis of variance (ANOVA) and Duncan’s multiple comparisons for nonparametric variables (*p* < 0.05).

## 3. Results

### 3.1. Weather Changes during the Maturation of Waxy Corn Kernels

**Figure 1:**
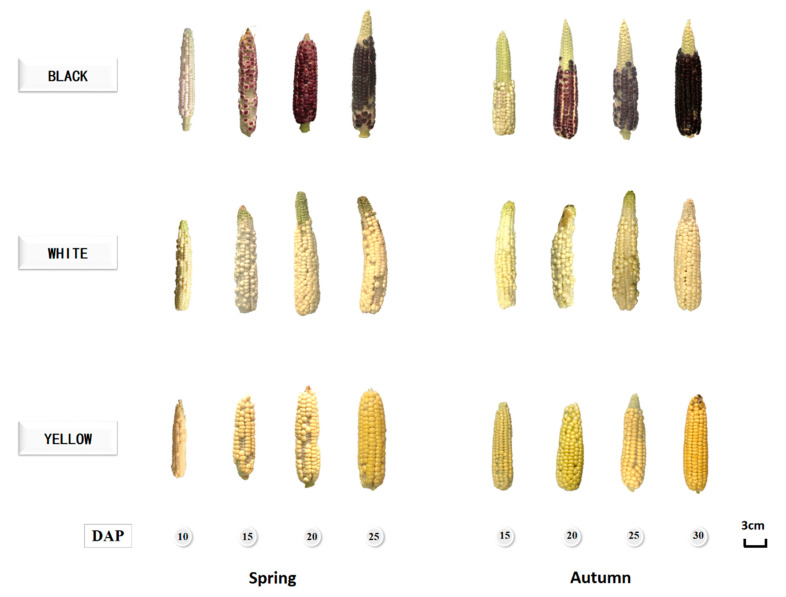
Pictures of waxy corns at different growing stages.

Figure 1 showed pictures of corn kernel development during maturation. It is obvious that the anthocyanin color had not been revealed in black waxy corn kernels at 10 and 15 DAP in both the spring and autumn seasons. As the kernels matured over time, anthocyanin gradually synthesized and accumulated in the aleurone layer, further revealing light purple in the coloring of the kernels. The corn cob increased in length and reached a plateau after 15 DAP, while the kernels continued to grow in size and darken in color until they fully matured at 25 DAP and 30 DAP in the spring and autumn, respectively. To be noticed, in black waxy corns, the kernels turned purple at 15 and 20 DAP in the spring and autumn, respectively. The coloring time of kernels in the autumn is about 5 days later than that in the summer, which is mainly caused by weather differences between the two seasons. In order to close the gap of the growing stage differences between the two seasons, the sampling time in the autumn is 5 days later than in the spring. Based on the taste and phenolics profiles discussed below, the best harvest time in the spring and autumn was 20 to 25 DAP and 25 to 30 DAP, respectively.

**Figure 2:**
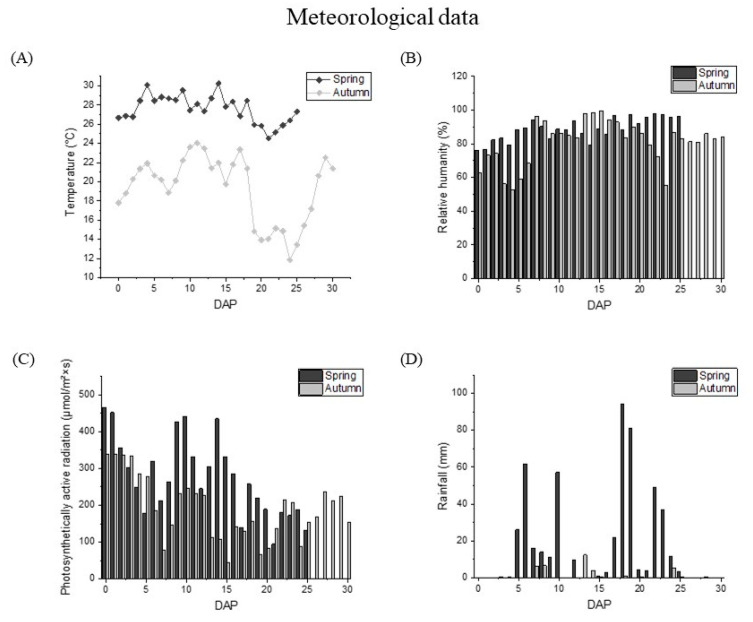
Meteorological data during kernel development. (**A**) Average temperatures (°C), (**B**) relative humidity (RH, %), (**C**) rainfall (mm), and (**D**) photosynthetically active radiation (PAR, μmol/m^2^·s). Data in spring and autumn were shown as black and grey, respectively.

The meteorological data of waxy corns from pollination to harvest in the summer and autumn were shown in Figure 2. The average temperature during the spring growing period was 27 °C, while the average temperature in the autumn was about 10 °C lower than that in the spring. Meanwhile, the temperature difference in the latter period of kernel development fluctuated acutely more than in the early stage of grain development, and the temperature difference within 5 days could reach more than 4 °C. The photosynthetic active radiation value in the summer was 286.60 μmol·m^−2^·s^−1^, which is significantly higher than that in the autumn (217.10 μmol·m^−2^·s^−1^). The rainfall value and relative humidity in the summer were also higher than those in the autumn. The rainfall in the summer can reach 17.53 mm, but only 0.97 mm in the autumn. The difference between relative humidity in the two seasons was also about 10%.

### 3.2. Phenolic and Anthocyanin Profiles Tested by HPLC

Content changes of various phenolic and anthocyanin components over the spring and autumn were shown in Table 1. Five types of phenolics including ferulic acid, gallic acid, lutin, *p*-coumaric acid, and epicatechin were identified in all three waxy corns. Two additional anthocyanins that were only detected in free phenolics extracts (cyanidin-3-*O*-glucoside and pelargonidin-3-*O*-glucoside, Cya-3-*O*-Glu, and Pal-3-*O*-Glu) were only found in black waxy corns.

The results show that different phenolics changed dramatically as the kernel matured. In black waxy corns, the two anthocyanins can only be detected until 20 DAP in both the spring and autumn, with contents between 0.5 mg/100 g DW and 1.0 mg/100 g DW. The contents in the autumn were higher than that in the spring. The content of Cya-3-*O*-Glu in the autumn is higher than in the summer, and the Pal-3-*O*-Glu showed a reverse changing trend with lower content. Epicatechin can only be detected as free forms and lutin was only available as bound fractions in all three types of waxy corns. Gallic acid, *p*-coumaric acid, and ferulic acid all existed as bound and free forms and except for gallic acid, the other two substances had much higher bound contents in three types of waxy corns in both seasons. By comparing the contents during kernel maturation, it is clear that almost all substances had the highest contents at 10 and 15 DAP in the spring and autumn, respectively, which hugely at 15 and 20 DAP. Phenolics in the autumn have a higher content than that in the summer for L1 and L2. L3 had little differences between the two seasons. Ferulic acid was the main bound phenolic in all three waxy corns in both seasons. L1 had a 6-fold higher ferulic acid in autumn with contents between 681.9 ± 55.7 and 1558 ± 35 mg/100 g DW and L3, on the other hand, had a slightly higher ferulic acid contents in the spring. It is remarkable that black waxy corns had much higher ferulic acid contents in the autumn than the other materials, followed by white waxy corns. *p*-Coumaric acid and lutin were also the main constituent in bound phenolics. The contents of *p*-coumaric acid and lutin in L1 and L2 are higher than that in L3, and both showed higher contents in the autumn than in the spring. Epicatechin and gallic acid were the main free components in all waxy corns. L1 and L2 had higher epicatechin contents for both seasons but L3 had higher gallic acid contents compared to epicatechin.

In order to integrally evaluate the total amount of anthocyanin in black waxy corns, glucoside attached to anthocyanin was removed by heat and acidification. The results were shown in Table 2. Cyanidin, pelargonidin, and peonidin were the main anthocyanins detected in black waxy corns, and cyanidin was the main component in the autumn. Pelargonidin had the highest content in the spring when the kernel matured. It is clear that anthocyanin started to accumulate at 15 and 20 DAP in the spring and autumn with contents of 35.7 ± 0.6 and 68.1 ± 0.8 mg/kg DW, and the content increased largely in the next 5 growing days. Then, the contents in the autumn dropped to 94.8 ± 0. 1 mg/kg DW at 30 DAP, while they kept growing in the spring to 175.6 ± 1.2 mg/kg DW. Comparing the constitution of anthocyanins between the two seasons, we can see that pelargonidin was affected greatly by weather conditions and the contents decreased hugely in the late stage of kernel development.

### 3.3. Changes of Relative Gene Expression Levels during Kernel Maturation

**Figure 3:**
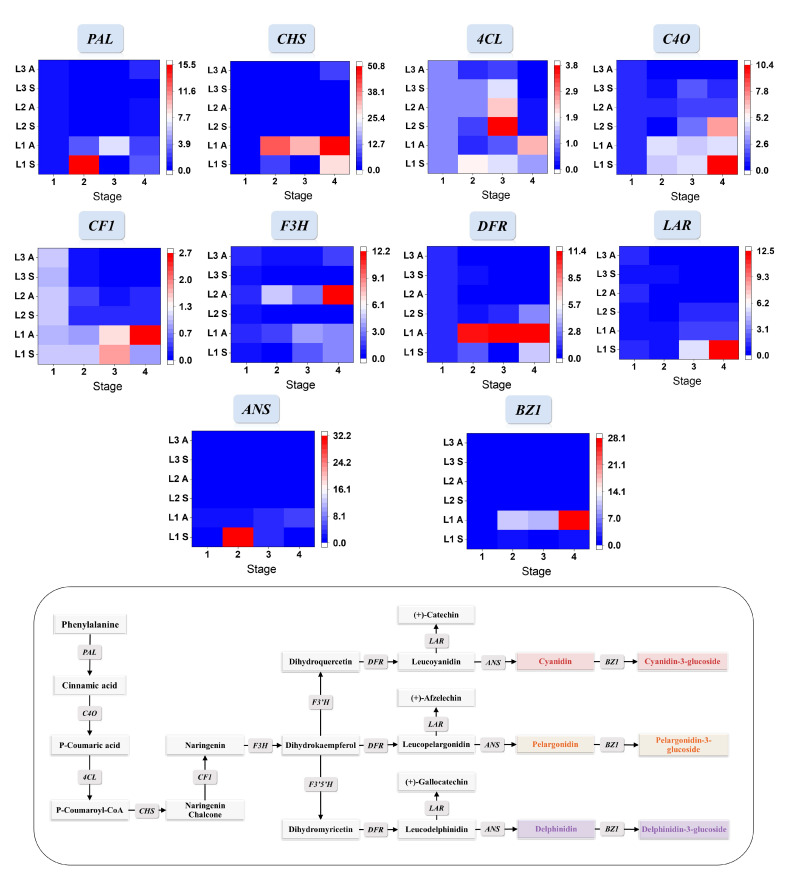
Related phenylpropanoid, flavonoid, and anthocyanin biosynthesis pathways, as well as relative gene expression level changes during kernel maturation, were presented in lines with gene ID labels on top. The relative gene expression levels were shown in heatmaps with a color gradient from blue to white to red representing expression levels from low to high. Stage 1 to 4 in the spring means 10, 15, 20, and 25 DAP; and the autumn means 15, 20, 25, and 30 DAP. S: spring; A: autumn.

The pathways controlling anthocyanin biosynthesis and the key gene expression levels of relative genes encoding the key enzymes in biosynthesis were shown in Figure 3. The gene expression levels of black, white, and yellow waxy corns were significantly different in the two seasons. In the spring, the expressions of *PAL* for L1 at 15 DAP increased significantly to 15 times of that at 10 DAP, and then they decreased to the lowest point at the next growing stage and elevated a bit at 30 DAP. In the autumn samples, the relative expression of *PAL* showed a continuous upward trend in the first two growing stages. *PAL* expressions in L2 and L3 had similar trends in both seasons by reducing growing time and had a small increase at the last growing stage. The expressions of *C4O* increased in both seasons, and the relative expression levels in the summer were higher than that in the autumn. *4CL* increased first and then decreased in the summer samples. In the autumn, the relative expression of *4CL* first reached the lowest point at 5 days and then increased significantly at the later stage of development. *CF1*, *F3H*, *DFR*, and *ANS*, as the regulatory genes closely related to anthocyanin synthesis, were highly expressed in L1 during kernel development, and the expression levels significantly increased in the coloring stage of black waxy corns (20 DAP in the autumn and 15 DAP in the summer). Compared to the relative expression levels in L1, *CF1*, *DFR*, and *ANS* of white and yellow waxy corns decreased significantly in both seasons during kernel development, especially the downstream genes that are critical to the formation and accumulation of anthocyanins, including *ANS* and *DFR*. The expression level of *BZ1* is related to the contents of anthocyanin with glucoside. Compared to L1 with L2 and L3, *BZ1* had higher relative expression levels in both seasons and increased as kernels matured.

### 3.4. In Vitro and Cell Antioxidant Activities

In vitro and cell antioxidant activities were shown in Table 3 and Table 4. The results showed that three types of waxy corns had high total ORAC values in both seasons with a range between 59.7 ± 0.1 and 234.0 ± 5.9 μmol QE/g DW. The total ORAC values in the autumn were higher than that in the spring, except for black waxy corns which decreased slightly in value in the autumn. In general, ORAC values decreased at 10 to 15 DAP and 15 to 20 DAP in the spring and autumn and then increased to reach a plateau in the latter stage of kernel development. The highest ORAC values are located at 10 and 15 DAP in the spring and autumn. The reduction in L2 and L3 was mainly caused by the decrease in both free and bound ORAC values. However, the free ORAC values in L1 were around 38.8 μmol TE/g DW at 10 and 15 DAP for the spring and autumn, lower than L2 and L3, but they maintained higher levels during kernel maturation compared to the other two waxy corns. The bound values were higher than the free ones for 1 to 2 times, which were in accordance with the phenolic results listed before. Especially during the suitable harvest period in summer (20 DAP), the bound ORAC values were 92.4 ± 3.0 μmol TE/g DW, 85.7 ± 0.9 μmol TE/g DW, and 94.8 ± 4.6 μmol TE/g DW for L1, L2 and L3, and about 2.5, 2.3, and 4.4 times of the free ORAC values, respectively. Compared the ORAC values in the harvest period in the spring and autumn in three waxy corns (20 and 25 DAP), the spring had a higher ORAC value compared to the autumn for about 10 to 50 μmol TE/g DW, and L1 had a higher antioxidant ability than L2 and L3 in the spring but the value reduced dramatically in the autumn during the harvest period. Hence, black waxy corn had better antioxidant ability in the spring than in the autumn.

The cell antioxidant abilities of three different waxy corns in the autumn were tested in order to have a rough comparison of the in vivo antioxidant abilities between the different colors of corns. In the no PBS wash protocol, the total CAA values of L1, L2, and L3 were significantly different among kernel maturation stages (*p* < 0.05). During the progress of kernel development, the total CAA values gradually decreased in three waxy corns, and the rate of reductions was different among varieties. The starting point of L1 was low with a CAA value of 82.0 ± 2.0 µmol QE/100 g at 15 DAP compared to L2 and L3 (115.7 ± 2.3 and 151.0 ± 4.3 µmol QE/100 g DW, respectively). However, unlike white and yellow waxy corns, black corns maintained a high level of CAA values of 45 µmol QE/100 g DW at the latter growing stages when L2 and L3 only had less than one-third of the amounts at 15 DAP. In L2, the total CAA value at 15 DAP was 115.7 ± 2.3 µmol QE/100 g DW, and the values reduced constantly to 16.7 ± 0.61 µmol QE/100 g DW at 30 DAP, with a difference of 7.91 times. L3 also decreased significantly with a difference of 3.66 times. During the suitable harvest period in the autumn, L3 had the highest CAA value of 52.6 ± 2.0 µmol QE/100 g DW, followed by L1.

In the PBS wash protocol, the total CAA values of the three waxy maize species were significantly lower than the values in the no PBS wash protocol. It was mainly caused by the decrease in CAA values in the bound forms compared with the free ones. During kernel development, the total CAA value also showed a similar decreasing trend as the no PBS protocol. The total CAA value of L1 had the least reduction, 1.4 folds, while L2 and L3 had a reduction of 8.6 and 8.3 folds compared to the values at 15 DAP and 35 DAP. L1 and L3 had a similar CAA value of 12.7 ± 0.6 and 19.3 ± 1.2 µmol QE/100 g DW, respectively, on the suitable harvest date (25 DAP), and L2 is significantly lower than the previous two.

## 4. Discussion

As introduced in the literature, the contents of bioactive compounds, including phenolics and anthocyanins, varied considerably depending on many factors such as agricultural practices, cultivars, and their characteristics [18,19]. Factors including growing area, temperature, and altitude during the growing period also had a strong relationship with content accumulation in plants [18]. Kernel nutrition accumulation was largely limited by cooler temperatures because of the decrease in plant vigor and essential enzymes [20]. The growing temperature was known to be positively correlated with maturation stages by delaying about 3 to 8 days of plant development per 1 °C of warmer temperature change in different flowers [21]. Therefore, the sampling time between the spring and autumn had a 5-day difference in order to eliminate the effect of the slow growth of waxy corn kernels in the autumn. Water deficit and low humidity would also affect the accumulation of metabolite in cereals [22], as well as the PAR level, which affected the distribution and biosynthesis of secondary metabolites in plants [23]. High accumulation of anthocyanin was seen in corns induced by UV radiation, cold temperature, and water stress [24]. Exposure to light may increase the number of flavonoids, and dry weather with low rainfall tends to have a higher number of total phenolics and anthocyanins in Chokeberries [25]. The anthocyanin contents during 15 to 20 DAP in the spring were lower compared to the autumn, but as the temperature in the autumn decreased, total anthocyanin reached the highest point at 25 DAP and decreased massively at 30 DAP, while contents in the spring accumulated along with time, of which pelargonidin had the biggest impact by different weather conditions compared to the other two. We could conclude that pelargonidin is more easily regulated by temperature and light intensity than other anthocyanins. As in the literature, severe stress conditions might inactivate antioxidant enzymes while increasing the biosynthesis of antioxidant flavonoids [26]. In our results, autumn tends to have higher phenolic contents with a lower growing temperature of around 20 °C. However, the rapid reduction in temperature at 20 to 25 DAP caused massive decreases in antioxidant abilities for all three corns.

The most common phenolic that existed in corn kernels are phenolic acids, flavonoids, and anthocyanin [27]. The total phenolic contents among different corn varieties, range from 243.8 to 320.1 mg GAE/100 g DW [28]. Corn has the highest phenolic content among wheat, oats, and rice [29]. In our results, all waxy corns had similar constitutions of phenolic profiles. However, the contents varied between corn varieties, and L1 seems to have better nutritional value considering phenolic contents and anthocyanins that are typically accumulated in pigment corns. Ferulic acid, which is usually combined with lignin in plants and is the main component of the cell wall, was normally the highest content in corn kernels, and its content of bound fraction was much higher than that of the free ones [29]. The content of ferulic acid in corn flour is about three times as high as wheat flour, 63 mg/kg, and the aleurone layer and the pericarp of grain usually contains 98% of the total ferulic acid [30]. According to Das et al.’s results, vanillic acid, caffeic acid, and *p*-coumaric acid were also the common phenolic acids found in corns. Most of the phenolics were existed in the corn pericarp, followed by the embryo and endosperm [31]. In pigmented corns, cyanidin, pelargonidin, and peonidin was detected in both seasons, which were also the main anthocyanin components in many pigmented corns reported [10,11,12]. Cya-3-Glu and Pg-3-Glu were the main anthocyanins in colored waxy corns and the value of cyanidin was about three times higher than pelargonidin, which is the same as our results, which found that cyanidin had relatively higher contents in black waxy corns compared to pelargonidin and peonidin. As in the literature, anthocyanin contents were much lower than phenolic acids and flavonoid, with a range from 2.2 to 291.5 AE µg/g DW, and the colored waxy corns contained higher phenolics than other common varieties (white and yellow), which was also in accordance with our results, which found that black waxy corns had higher contents of ferulic acid and other flavonoids [32]. Except for anthocyanins, many flavonoids, including rutin, kaempferol, quercetin, naringin, hesperidin, and their derivatives are the most commonly reported flavonoids [33]. Rutin and epicatechin were also detected in our result and had high contents in black waxy corns compared to the other two.

Generally, bound phenolic contents are the dominant factor for the antioxidant capacity level compared to the free ones because the phenolic was known to be the important antioxidant in waxy corns and the bound phenolics contents were much higher than the free ones, which is also shown in the free and bound ORAC values. The content of ORAC and CAA values also had similar trends during kernel maturation. CAA quantifies the antioxidant activity of samples by detecting the change of fluorescence intensity over a period of time and can identify the bioavailability, absorption, distribution, and metabolism of antioxidant substances. Wash and no-wash protocols were tested to determine the absorption degree of antioxidants to the cell. According to the polarity, the solubility and molecular size of the antioxidants can vary the degree of absorption by the cell, and hence determine whether the substances were to react with the oxygen radical by entering the cells or react by combining with the cell membrane outside the cell [34]. There were significant differences in cell absorptivity among different types of waxy corns. In free CAA values, except for black waxy corns, white and yellow waxy corns had a high absorption ratio of 44% and 74% at 15 DAP and remained at a high ratio of around 40 to 50% during the following kernel maturation. In black waxy corns, the ratio of cell absorptivity was low at 15 DAP and increased to a high level of 65% at 20 to 25 DAP when the anthocyanin content increased. Though anthocyanin was hard to be absorbed by the cell because of its positive atom, it may improve the efficiency of cell absorption for other antioxidants and then increase the CAA wash values in L1. The cell antioxidant abilities varied among varieties which means that different constitutions of phenolic contents had different antioxidant capacities.

Rt-qPCR results of gene expressions that encode the relevant enzymes in the anthocyanin biosynthesis were analyzed to interpret the difference between phenolic profiles in three waxy corns. It is known that genes *PAL*, *C4O*, and *4CL* had similar expression profiles with the downstream genes involved in the anthocyanin pathway in pigmented tissues [5]. From our results, these genes in black waxy corns had a similar changing pattern that increased downstream genes in flavonoid synthesis compared to L2 and L3 during kernel development. Upregulated gene expressions in *PAL* and *C4O* could also increase the substrate for lignin generation and, therefore, cause higher phenolic contents, such as ferulic acid. *CF1*, *F3H*, and *DFR* are all key genes in the anthocyanin biosynthesis pathway, and a low expression of *CF1* would lead to the accumulation of chalcones, which would turn the color of plants yellow [35]. *4CL* is a key gene encoding 4-coumarate: CoA ligase used to affect the synthesis of 4-coumaryl CoA, which is an important limiting intermediate, before branching to synthesize aromatic volatiles, including coumarin and lignin [36]. *CHS* is an important gene to promote the synthesis of flavonoids, which could also affect the accumulation of anthocyanin [37]. Its relative expression increased with kernel development, indicating that the flavonoid synthesis pathway in black waxy maize maintained high efficiency. The mutation of *CHS* was one of the most common causes of color loss in kernels [38]. *CF1*, *F3H*, and *DFR* are key genes involved in the anthocyanin biosynthesis pathway, and a low expression of *CF1* will lead to the accumulation of chalcone products and make the plant color yellow [35]. The enzymes encoded by *F3H*, *CHS*, and *CF1* worked together to regulate the formation of downstream products [39]. As a key gene for the synthesis of corresponding colorless anthocyanins, the expression specificity of *DFR* and its substrate specificity would lead to a change in flower color. In plant tissues with high *DFR* expression, anthocyanins would accumulate in large quantities, while tissues with low *DFR* expression would produce a large number of anthocyanins instead of colorless anthocyanins [40,41,42]. These genes had higher relative expression levels in L1 compared to the other two waxy corns. Previous studies showed that an increase in the expression of *LAR* can promote the proanthocyanin pathway, thereby reducing anthocyanin levels, and *ANS* has also been found to affect anthocyanin coloring [43]. In our results, *LAR* expression was low in black waxy corns between the first and second stages in both the spring and autumn, and anthocyanin started to accumulate. The expressions of *ANS* also increased slowly as the corn matured and anthocyanin accumulation continued. *BZ1* encoding anthocyanin glycosyltransferase has no correlation with anthocyanin or flavonol accumulation [44]. Overall, the gene expression level could, to some extent, affect the accumulation of phenolic contents. Hence, we illustrated that these genes were highly related to anthocyanin biosynthesis. Meanwhile, previous literature had shown that low expression of *DFR* and *ANS* could lead to major pigment loss, and *DFR* is highly specific with respect to substrate and could affect the composition and coloring of anthocyanins [7].

## 5. Conclusions

After evaluating the phenolic and anthocyanin profiles, as well as antioxidant capacities of three waxy corns under the impact of essential enzymes and different weather conditions, we know that ferulic acid was the main phenolic content in different colors of waxy corns and that cyanidin was the main anthocyanin component in black waxy corns. The bound fraction of phenolics was higher than the free ones. The accumulation of anthocyanin was affected by weather conditions and the differential expression of related genes during kernel development. *DFR*, *CF1*, and *ANS*, as regulatory genes of anthocyanin biosynthesis, showed increased expression levels during kernel maturation in the two seasons. Our results provided information for the cultivation of waxy corns and further illustrated the potential of colored waxy corns as a valuable addition to anthocyanin intake.

## Figures and Tables

**Table 1:** Phytochemical content identified in waxy corns during kernel maturation. Values are shown as means ± SD (*n* = 3).

(mg/100 g DW)				Spring				Autumn		
	DAP		10	15	20	25	15	20	25	30
L1	Cya-3-*O*-Glu	F	ND	ND	0.3 ± 0.0 ^a^	0.7 ± 0.1 ^b^	ND	0.9 ± 0.1 ^a^	2.4 ± 0.1 ^c^	1.8 ± 0.3 ^b^
	Pal-3-*O*-Glu	F	ND	ND	0.4 ± 0.1 ^c^	1.3 ± 0.1 ^d^	ND	0.6 ± 0.0 ^a^	0.9 ± 0.1 ^b^	1.0 ± 0.1 ^c^
	Epicatechin	F	74.8 ± 4.2 ^b^	72.6 ± 0.9 ^b^	41.5 ± 0.5 ^a^	38.5 ± 0.4 ^a^	247.0 ± 0.2 ^d^	140.1 ± 1.9 ^c^	91.5 ± 0.3 ^b^	87.0 ± 3.6 ^a^
	Lutin	B	30.5 ± 4.1 ^b^	14.4 ± 1.8 ^a^	13.5 ± 3.2 ^a^	15.1 ± 2.1 ^a^	92.1 ± 1.3 ^c^	37.8 ± 2.3 ^a^	36.2 ± 1.8 ^a^	50.8 ± 3.1 ^b^
	Gallic acid	F	41.2 ± 0.6 ^d^	21.0 ± 0.2 ^c^	15.7 ± 1.2 ^b^	12.4 ± 0.4 ^a^	43.1 ± 0.9 ^c^	20.3 ± 0.6 ^b^	18.8 ± 1.6 ^b^	15.3 ± 2.5 ^a^
		B	2.8 ± 0.5 ^b^	1.1 ± 0.1 ^a^	1.0 ± 0.2 ^a^	1.1 ± 0.0 ^a^	18.6 ± 0.7 ^c^	10.0 ± 0.3 ^a^	13.6 ± 0.3 ^b^	27.7 ± 1.6 ^d^
		T	44.0 ± 0.2 ^d^	22.1 ± 0.2 ^c^	16.7 ± 1.1 ^b^	13.5 ± 0.4 ^a^	61.6 ± 1.6 ^c^	30.3 ± 0.8 ^a^	32.4 ± 1.5 ^a^	43.0 ± 1.4 ^b^
	*p*-Coumaric acid	F	7.4 ± 0.4 ^c^	2.5 ± 0.1 ^b^	2.2 ± 0.1 ^ab^	1.9 ± 0.1 ^a^	7.3 ± 0.2 ^d^	2.4 ± 0.3 ^c^	1.1 ± 0.0 ^a^	1.6 ± 0.1 ^b^
		B	26.1 ± 3.8 ^c^	6.8 ± 0.2 ^a^	14.0 ± 2.4 ^b^	4.0 ± 0.2 ^a^	92.2 ± 3.3 ^c^	46.8 ± 7.5 ^b^	8.7 ± 0.5 ^a^	9.2 ± 1.0 ^a^
		T	33.5 ± 3.4 ^c^	9.4 ± 0.4 ^a^	16.2 ± 2.4 ^b^	5.9 ± 0.2 ^a^	99.5 ± 3.3 ^c^	49.2 ± 7.4 ^b^	9.8 ± 0.5 ^a^	10.8 ± 1.0 ^a^
	Ferulic acid	F	5.7 ± 0.2 ^c^	2.7 ± 0.1 ^b^	2.8 ± 0.1 ^b^	2.4 ± 0.1 ^a^	3.8 ± 0.1 ^c^	1.9 ± 0.1 ^b^	1.6 ± 0.1 ^a^	1.6 ± 0.1 ^a^
		B	249.4 ± 30.0 ^b^	158.6 ± 14.4 ^a^	259.9 ± 43.4 ^b^	234.9 ± 25.9 ^b^	1554 ± 35 ^b^	680.0 ± 55.7 ^a^	713.7 ± 64.2 ^a^	815.3 ± 108 ^a^
		T	255.1 ± 30.0 ^b^	161.4 ± 14.5 ^a^	262.7 ± 43.4 ^b^	237.3 ± 26.0 ^b^	1558 ± 35 ^b^	681.9 ± 55.7 ^a^	715.3 ± 64.3 ^a^	817.0 ± 108 ^a^
L2	Epicatechin	F	78.2 ± 1.1 ^d^	40.4 ± 0.6 ^c^	22.4 ± 1.6 ^b^	11.8 ± 0.5 ^a^	144.5 ± 8.9 ^b^	204.2 ± 4.5 ^c^	142.8 ± 3.2 ^b^	101.8 ± 9.5 ^a^
	Lutin	B	23.8 ± 0.8 ^c^	22.3 ± 0.4 ^b^	19.1 ± 1.0 ^a^	20.0 ± 0.2 ^a^	47.9 ± 1.0 ^d^	25.5 ± 0.6 ^c^	22.3 ± 1.5 ^b^	14.9 ± 2.3 ^a^
	Gallic acid	F	48.1 ± 7.1 ^b^	20.3 ± 3.2 ^a^	19.9 ± 1.3 ^a^	14.65 ± 0.7 ^a^	27.2 ± 2.7 ^c^	22.6 ± 2.4 ^b^	14.9 ± 1.4 ^a^	15.4 ± 0.0 ^a^
		B	11.1 ± 0.7 ^b^	1.5 ± 0.1 ^a^	1.5 ± 0.2 ^a^	1.45 ± 0.1 ^a^	4.2 ± 0.3 ^b^	1.8 ± 0.2 ^a^	1.7 ± 0.0 ^a^	1.5 ± 0.2 ^a^
		T	59.3 ± 7.8 ^b^	21.7 ± 3.1 ^a^	21.3 ± 1.0 ^a^	16.10 ± 0.7 ^a^	31.4 ± 1.8 ^c^	24.4 ± 2.3 ^b^	16.6 ± 1.4 ^a^	16.9 ± 0.2 ^a^
	*p*-Coumaric acid	F	7.2 ± 0.2 ^d^	3.0 ± 0.1 ^c^	1.7 ± 0.0 ^b^	1.4 ± 0.0 ^a^	16.2 ± 2.6 ^c^	18.1 ± 0.2 ^d^	2.7 ± 0.0 ^b^	1.2 ± 0.1 ^a^
		B	20.8 ± 2.8 ^b^	27.3 ± 1.3 ^c^	6.2 ± 0.6 ^a^	5.3 ± 0.3 ^a^	61.8 ± 2.7 ^d^	14.3 ± 0.3 ^c^	12.0 ± 1.0 ^b^	4.3 ± 0.5 ^a^
		T	28.0 ± 1.8 ^b^	30.3 ± 1.4 ^c^	7.9 ± 0.6 ^a^	6.7 ± 0.3 ^a^	78.1 ± 3.3 ^d^	32.3 ± 0.4 ^c^	14.8 ± 1.0 ^b^	5.4 ± 0.5 ^a^
	Ferulic acid	F	7.0 ± 0.4 ^c^	3.3 ± 0.1 ^b^	2.2 ± 0.3 ^a^	2.4 ± 0.1 ^a^	5.6 ± 0.9 ^c^	2.0 ± 0.1 ^b^	1.5 ± 0.1 ^ab^	0.9 ± 0.1 ^a^
		B	192.8 ± 33.5 ^a^	176.9 ± 19.4 ^a^	184.2 ± 27.5 ^a^	190.8 ± 21.3 ^a^	606.9 ± 36.0 ^d^	405.0 ± 8.2 ^c^	344.3 ± 39.9 ^b^	198.3 ± 30.4 ^a^
		T	199.9 ± 33.8 ^a^	180.2 ± 19.6 ^a^	186.4 ± 27.7 ^a^	193.2 ± 15.0 ^a^	612.4 ± 25.8 ^d^	407.0 ± 8.3 ^c^	345.8 ± 39.9 ^b^	199.2 ± 21.6 ^a^
L3	Epicatechin	F	12.6 ± 1.3 ^c^	8.5 ± 0.1 ^b^	5.5 ± 0.4 ^a^	5.0 ± 0.7 ^a^	17.9 ± 0.5 ^c^	19.1 ± 1.1 ^c^	15.0 ± 0.8 ^b^	8.0 ± 0.4 ^a^
	Lutin	B	37.0 ± 5.7 ^c^	20.9 ± 3.1 ^b^	11.4 ± 2.2 ^a^	11.9 ± 1.2 ^a^	25.8 ± 1.0 ^c^	14.0 ± 0.4 ^b^	10.2 ± 0.6 ^a^	10.5 ± 1.1 ^a^
	Gallic acid	F	37.6 ± 1.2 ^d^	18.3 ± 0.1 ^b^	13.4 ± 2.0 ^a^	21.3 ± 0.6 ^c^	44.7 ± 0.9 ^c^	30.9 ± 1.8 ^b^	14.5 ± 0.5 ^a^	17.0 ± 2.4 ^a^
		B	2.2 ± 0.0 ^c^	1.5 ± 0.1 ^a^	1.3 ± 0.1 ^a^	1.51 ± 0.1 ^ab^	1.6 ± 0.1 ^b^	0.6 ± 0.0 ^a^	0.5 ± 0.1 ^a^	0.5 ± 0.0 ^a^
		T	39.9 ± 0.8 ^d^	19.7 ± 0.1 ^b^	14.7 ± 2.1 ^a^	22.79 ± 0.7 ^c^	46.2 ± 4.2 ^c^	31.5 ± 1.8 ^b^	15.0 ± 0.5 ^a^	17.6 ± 1.7 ^a^
	*p*-Coumaric acid	F	7.9 ± 1.3 ^b^	2.3 ± 0.2 ^a^	1.8 ± 0.0 ^a^	1.8 ± 0.0 ^a^	11.3 ± 0.6 ^c^	3.0 ± 0.1 ^b^	1.5 ± 0.1 ^a^	1.3 ± 0.0 ^a^
		B	17.3 ± 1.5 ^d^	11.1 ± 0.5 ^c^	5.4 ± 0.9 ^a^	7.3 ± 0.3 ^b^	18.8 ± 0.7 ^c^	14.1 ± 0.8 ^b^	5.2 ± 1.0 ^a^	4.1 ± 0.4 ^a^
		T	25.2 ± 2.8 ^c^	13.4 ± 0.6 ^b^	7.1 ± 0.7 ^a^	9.1 ± 0.3 ^a^	30.1 ± 1.2 ^c^	17.1 ± 0.9 ^b^	6.7 ± 0.9 ^a^	5.4 ± 0.4 ^a^
	Ferulic acid	F	9.1 ± 0.4 ^c^	3.8 ± 0.0 ^b^	2.6 ± 0.2 ^a^	2.6 ± 0.3 ^a^	ND	3.2 ± 0.3 ^b^	1.9 ± 0.1 ^a^	1.7 ± 0.4 ^a^
		B	306.7 ± 38.9 ^a^	286.4 ± 20.8 ^a^	263.8 ± 23.8 ^a^	382.8 ± 8.9 ^b^	162.5 ± 14.0 ^b^	108.7 ± 8.9 ^a^	78.7 ± 10.3 ^a^	77.3 ± 21.9 ^a^
		T	315.8 ± 39.0 ^b^	290.1 ± 20.8 ^ab^	266.4 ± 24.0 ^a^	385.4 ± 8.6 ^c^	162.5 ± 14.0 ^b^	111.9 ± 9.2 ^a^	80.63 ± 7.3 ^a^	78.97 ± 22.3 ^a^

Different small letters in the upper right corner of the values in the same row indicate a statistically significant difference (*p* < 0.05). ND: not detectable. F: free; B: bound; T: total.

**Table 2:** Anthocyanin contents identified after hydrolysis reaction in black waxy corns during kernel maturation. Values are presented as means ± SD (*n* = 3).

Anthocyanin (mg/kg DW)	Spring				Autumn			
	10 DAP	15 DAP	20 DAP	25 DAP	15 DAP	20 DAP	25 DAP	30 DAP
Cyanidin	ND	14.7 ± 0.3 ^a^	23.8 ± 0.9 ^b^	74.5 ± 0.3 ^c^	ND	50.4 ± 0.2 ^a^	111.1 ± 0.9 ^c^	76.4 ± 0.3 ^b^
Pelargonidin	ND	17.3 ± 0.3 ^a^	25.8 ± 0.8 ^b^	97.6 ± 1.1 ^c^	ND	13.5 ± 0.5 ^a^	21.2 ± 0.8 ^c^	15.4 ± 0.3 ^b^
Peonidin	ND	3.7 ± 0.0 ^b^	3.6 ± 0.0 ^b^	3.4 ± 0.1 ^a^	ND	4.2 ± 0.2 ^b^	4.2 ± 0.0 ^b^	3.0 ± 0.1 ^a^
Total	ND	35.7 ± 0.6 ^a^	53.2 ± 0.7 ^b^	175.6 ± 1.2 ^c^	ND	68.1 ± 0.8 ^a^	136.5 ± 0.2 ^c^	94.8 ± 0.1 ^b^

Different small letters in the upper right corner of the values in the same row indicate a statistically significant difference (*p* < 0.05). ND: not detectable.

**Table 3:** ORAC values of three waxy corns during kernel development. Values are presented as means ± SD (*n* = 3).

				Spring				Autumn		
	DAP		10	15	20	25	15	20	25	30
ORAC (μmol TE/g DW)	L1	F	38.8 ± 2.3 ^c^	29.9 ± 2.1 ^ab^	26.0 ± 1.7 ^a^	33.3 ± 4.3 ^b^	38.1 ± 1.5 ^c^	24.8 ± 0.5 ^b^	19.3 ± 0.1 ^a^	23.6 ± 0.8 ^b^
		B	102.6 ± 8.1 ^c^	52.9 ± 0.6 ^a^	92.4 ± 3.0 ^c^	77.9 ± 1.6 ^b^	97.9 ± 4.7 ^d^	35.1 ± 0.6 ^a^	46.8 ± 3.8 ^b^	64.1 ± 2.9 ^c^
		T	141.4 ± 9.4 ^c^	82.8 ± 11.9 ^a^	118.4 ± 4.4 ^b^	111.2 ± 3.6 ^b^	136.0 ± 5.5 ^c^	59.9 ± 0.1 ^a^	66.1 ± 4.2 ^a^	87.7 ± 3.6 ^b^
	L2	F	66.7 ± 0.3 ^b^	40.4 ± 13.4 ^a^	26.7 ± 0.3 ^a^	29.1 ± 5.9 ^a^	40.4 ± 0.2 ^d^	23.0 ± 2.5 ^c^	16.4 ± 2.1 ^c^	13.8 ± 0.8 ^c^
		B	92.6 ± 7.0 ^b^	87.0 ± 6.5 ^ab^	85.7 ± 0.9 ^ab^	77.3 ± 5.8 ^a^	193.6 ± 6.1 ^e^	117.2 ± 1.2 ^a^	86.0 ± 9.1 ^b^	56.1 ± 3.6 ^d^
		T	159.2 ± 7.3 ^c^	127.3 ± 19.2 ^b^	112.4 ± 1.2 ^ab^	106.4 ± 0.1 ^a^	234.0 ± 5.9 ^d^	140.2 ± 2.6 ^a^	102.3 ± 9.3 ^a^	70.0 ± 4.3 ^c^
	L3	F	39.1 ± 2.0 ^c^	19.0 ± 3.1 ^ab^	17.7 ± 1.0 ^a^	22.1 ± 1.6 ^b^	44.3 ± 3.8 ^b^	20.7 ± 2.7 ^a^	21.2 ± 1.4 ^a^	16.4 ± 1.4 ^a^
		B	122.8 ± 8.6 ^c^	96.6 ± 10.4 ^ab^	94.8 ± 4.6 ^a^	115.2 ± 13.8 ^bc^	187.1 ± 11.0 ^c^	114.4 ± 8.5 ^b^	76.8 ± 1.1 ^a^	94.9 ± 14.0 ^a^
		T	161.9 ± 10.5 ^c^	115.6 ± 12.1 ^a^	112.5 ± 3.7 ^a^	137.3 ± 15.0 ^b^	231.4 ± 10.6 ^c^	135.0 ± 9.2 ^b^	97.9 ± 2.5 ^a^	111.3 ± 15.2 ^a^

Different small letters in the upper right corner of the values in the same row indicate a statistically significant difference (*p* < 0.05). F: free; B: bound; T: total.

**Table 4:** CAA values of three waxy corns during kernel development in the autumn. Values are presented as means ± SD (*n* = 3).

	DAP		15	20	25	30
L1	CAA value (μmol QE/100 g DW, no PBS wash)	F	17.3 ± 1.1 ^b^	14.9 ± 0.8 ^a^	19.6 ± 0.7 ^c^	15.5 ± 1.3 ^ab^
		B	64.7 ± 2.9 ^c^	32.7 ± 0.8 ^b^	28.6 ± 1.8 ^a^	25.0 ± 1.8 ^a^
		T	82.0 ± 2.0 ^c^	47.6 ± 1.3 ^b^	48.3 ± 2.2 ^b^	40.5 ± 0.7 ^a^
	CAA value (μmol QE/100 g DW, PBS wash)	F	3.4 ± 1.1 ^a^	9.7 ± 0.7 ^b^	12.7 ± 0.5 ^c^	4.4 ± 0.4 ^a^
		B	31.2 ± 1.1 ^c^	6.4 ± 0.7 ^a^	6.3 ± 0.2 ^a^	8.3 ± 0.3 ^b^
		T	34.6 ± 1.1 ^d^	16.0 ± 1.3 ^b^	19.0 ± 0.7 ^c^	12.7 ± 0.6 ^a^
L2	CAA value (μmol QE/100 g DW, no PBS wash)	F	49.9 ± 2.8 ^c^	12.2 ± 0.2 ^b^	7.8 ± 0.8 ^a^	7.2 ± 0.4 ^a^
		B	65.8 ± 2.7 ^d^	56.8 ± 1.7 ^c^	21.2 ± 2.1 ^b^	9.5 ± 0.2 ^a^
		T	115.7 ± 2.3 ^d^	69.0 ± 1.7 ^c^	29.0 ± 2.1 ^b^	16.7 ± 0.6 ^a^
	CAA value (μmol QE/100 g DW, PBS wash)	F	22.1 ± 0.9 ^c^	7.1 ± 0.5 ^b^	4.1 ± 0.8 ^a^	3.8 ± 0.2 ^a^
		B	21.8 ± 1.0 ^d^	14.9 ± 2.5 ^c^	5.4 ± 1.1 ^b^	2.1 ± 0.4 ^a^
		T	43.9 ± 1.8 ^d^	22.0 ± 2.6 ^c^	9.6 ± 1.3 ^b^	5.9 ± 0.3 ^a^
L3	CAA value (μmol QE/100 g DW, no PBS wash)	F	79.1 ± 2.4 ^b^	32.3 ± 1.9 ^a^	32. 7 ± 0.2 ^a^	33.8 ± 1.1 ^a^
		B	71.9 ± 3.5 ^c^	29.9 ± 1.8 ^b^	19.9 ± 1.9 ^a^	17.6 ± 1.2 ^a^
		T	151.0 ± 4.3 ^c^	62.1 ± 1.0 ^b^	52.6 ± 2.0 ^a^	51.4 ± 0.6 ^a^
	CAA value (μmol QE/100 g DW, PBS wash)	F	58.6 ± 0.8 ^d^	15.2 ± 0.0 ^c^	10.2 ± 0.9 ^a^	13.9 ± 0.4 ^b^
		B	30.8 ± 1.5 ^c^	12.3 ± 1.8 ^b^	5.0 ± 0.6 ^a^	5.4 ± 0.8 ^a^
		T	89.4 ± 2.2 ^d^	27.5 ± 1.8 ^c^	15.2 ± 1.4 ^a^	19.3 ± 1.2 ^b^

Different small letters in the upper right corner of the values in the same row indicate a statistically significant difference (*p* < 0.05). ND: not detectable. F: free; B: bound; T: total.

## Data Availability

Data is contained within the article and supplementary material.

## Supplementary Materials

The following supporting information can be downloaded at https://www.mdpi.com/article/10.3390/foods12071486/s1, Table S1: Primer sequence of genes related to enzymes in phenolic and flavonoid biosynthesis pathways.
