# Supplementary material for: The Accumulation and Biosynthesis of Anthocyanin in Black, White, and Yellow Waxy Corns (Zea mays L. sinensis kulesh) during Kernel Maturation
Source: Foods. 2023 Apr 1;12(7):1486. doi: 10.3390/foods12071486 (PMC10094669; doi:10.3390/foods12071486)
Supplement: Supplementary file 1 [file foods-12-01486-s001.zip › foods-2258398-supplementary.pdf]

Supplemental Table S1. Primer sequence of genes related to enzymes in phenolic and flavonoid biosynthesis pathways.

Gene name	GeneID	Primer Sequence (5'-3')	
4CL	103653401	Forward	CGAGCAAGACTTGGACTTCG
		Reverse	ATCAGGATGGTGTTGAGCGA
PAL	100281042	Forward	GGATGGTGGAGGAGTACAGG
		Reverse	CGGCATTGAGGAATCGGATG
C4O	100272801	Forward	TGTTCCGCATCATGTTCGAC
		Reverse	GTTGTACTCGAAGCTCTGCG
CHS	100282642	Forward	ATGATCCGGAAGCGGTACAT
		Reverse	TGGTACATCATCAGGCGGTT
BZ1	732800	Forward	TCGTCTTGCTGTTCAGAGGT
		Reverse	CCAAAGCCAAGGTACAGAAGG
CF1	100284018	Forward	AGCTTCCTCCTCCTCACCTA
		Reverse	GTCTCCGAGCCCATTTTCAC
DFR	100286107	Forward	GCCGAGATCTTCCTCTTCGA
		Reverse	CTGGAGCTTCTTGGACGAGA
F3H	542712	Forward	CGTTGTTATTGCTGCGTGTG
		Reverse	TTACAATGCACTCCGGTCCA
ANS	100285776	Forward	TTCAAAAGTCCGGTGCACAG
		Reverse	TCTGTCCTTCCGCAATTCCT
LAR	100282500	Forward	GCCTTGCAACTTTGAGCTCT
		Reverse	CGAAACTTGGAGAGCTGTCG
ADF	100232871	Forward	TCGTCTTGCTGTTCAGAGGT
		Reverse	CCAAAGCCAAGGTACAGAAGG
